# *Akkermansia* deficiency and mucin depletion are implicated in intestinal barrier dysfunction as earlier event in the development of inflammation in interleukin-10-deficient mice

**DOI:** 10.3389/fmicb.2022.1083884

**Published:** 2023-01-09

**Authors:** Beatriz López-Cauce, Marta Puerto, Juan José García, Manuel Ponce-Alonso, Federico Becerra-Aparicio, Rosa del Campo, Isabel Peligros, María J. Fernández-Aceñero, Yésica Gómez-Navarro, José M. Lara, José Miranda-Bautista, Ignacio Marín-Jiménez, Rafael Bañares, Luis Menchén

**Affiliations:** ^1^Servicio de Aparato Digestivo, Hospital General Universitario Gregorio Marañón, Instituto de Investigación Sanitaria Gregorio Marañón, Madrid, Spain; ^2^Centro de Investigación Biomédica en Red de Enfermedades Hepáticas y Digestivas (CIBEREHD), Madrid, Spain; ^3^Departamento de Microbiología y Parasitología Facultad de Farmacia, Universidad Complutense, Madrid, Spain; ^4^Servicio de Microbiología, Hospital Ramón y Cajal, CIBERINFEC, IRYCIS, Madrid, Spain; ^5^Servicio de Anatomía Patológica, Hospital General Universitario Gregorio Marañón, Madrid, Spain; ^6^Departamento de Medicina, Facultad de Medicina, Universidad Complutense, Madrid, Spain

**Keywords:** *Akkermansia muciniphila*, gut microbiome, mucin, intestinal barrier, interleukin-10-deficient mice, inflammatory bowel disease

## Abstract

**Background:**

Dysbiosis and mucin depletion are related with intestinal barrier dysfunction and seems to be an early pathophysiological event in inflammatory bowel disease (IBD). The objective of this work is to study these parameters in the natural history of colitis in IL-10 deficient mice (IL-10^−/−^).

**Methods:**

Wild type (WT) and IL-10^−/−^. mice were followed until sacrifice at 3, 5, 10, 20, 57, and 70 weeks. Body weight, colonic weight/length ratio and *in vivo* intestinal permeability were registered. Expression of inflammatory and adhesion molecules in the colon was explored by qPCR as *Mucin-2* (*MUC-2*) and molecules involved in goblet cell maturation *Interleukin-18 (IL-18)* and *WAP Four-Disulfide Core Domain 2 (WFDC2)*, the endoplasmic reticulum stress markers *X-box-binding protein (Xbp-1)* and *Reticulon-4B (RTN-4B)*. Bacterial composition in feces and colonic mucosa was determined by massive sequencing of the V3–V4 regions of 16S rDNA gene.

**Results:**

IL-10^-/-^ mice showed histological inflammation at weeks 20 and 57, but most notably the intestinal permeability was significantly higher from week 10. Concordantly, the number of goblet cells and expression of *MUC-2, IL-18, WFDC2* and *Xbp-1* were significantly lower in KO from week 10. Nevertheless, no significant differences were found in the mRNA expression of *MUC-2* or *Xbp-1* between both groups—derived colon organoids. Significant bacterial differences began at week 5, being the *Akkermansia* deficiency in KO the most relevant result.

**Conclusion:**

Gut microbiota alterations and mucin depletion are associated with early intestinal barrier dysfunction and precede overt gut inflammation in this animal model of IBD.

## 1. Introduction

Since the spontaneous development of chronic intestinal inflammation in mice deficient in interleukin-10 (IL-10^−/−^) was described more than two decades ago, this experimental model is one of the major approximations in the study of inflammatory bowel disease (IBD) pathophysiology (Kuhn et al., 1993). Its relevance was outlined, even more, after the report of children diagnosed with early-onset Crohn’s disease (CD) who carried mutations in both *IL-10* and *IL-10 receptor (IL-10R)* genes (Glocker et al., 2009, 2010). IL-10 exerts a pivotal role in the maintenance of mucosal tolerance, thus, in the absence of its suppressive effect, IL-10 deficiency leads to sustained small bowel and colonic inflammation due to an excessive production of proinflammatory cytokines by activated macrophages and T cells (Berg et al., 1996; Kaser et al., 2010).

The chronological course of the intestinal inflammatory process in IL-10 deficient mice has been already described (Kuhn et al., 1993; Berg et al., 1996), although there are substantial variations in the inflammation severity depending on mice backgrounds and housing conditions (Kobayashi et al., 2003; Noguchi et al., 2009). Weight loss and anemia start at 4–8 weeks of age. The inflammatory infiltrate affects both mucosa and submucosa and is composed by lymphocytes, immunoglobulin A (IgA) positive plasma cells, macrophages, neutrophils and eosinophils; inflammation is associated with disturbances of the mucosal architecture such as enlarged and branched crypts, thickening of the basement membrane, and superficial erosions. In addition, the implication of the microbiota on the pathogenesis of colitis in IL-10 deficient mice is supported by solid evidences such as the fact that inflammation is more severe in mice kept under conventional housing conditions, in comparison to animals maintained under specific pathogen-free and germ-free conditions (Berg et al., 1996; Sellon et al., 1998; Spencer et al., 2002).

The dysfunction of the intestinal barrier usually overlaps with the stimulation of mucosal immune cells by luminal antigens, which is a critical pathophysiological event in IBD, both in human and in experimental models, including IL-10 deficient mice (Shouval et al., 2014; Keubler et al., 2015). Intestinal epithelial cells (IECs) constitute the main cellular element of the intestinal barrier between the mucosal immune system and external milieu (Turner, 2006; Groschwitz and Hogan, 2009; Turner, 2009). A functional barrier and IECs apical-basal polarity are built up and maintained through specialized plasma membrane structures, containing adhesive and scaffolding proteins known as the apical junction complexes (AJCs; Weisz and Rodriguez-Boulan, 2009). These complexes seal the paracellular space between adjacent IECs, and are mainly composed by tight junctions (TJs): Zonula Occludens (ZO), Occludins, Claudins, etc. and adherent junctions (AJs): E-Cadherin, members of Nectin family, Catenins, etc. (Bhat et al., 2019; Nighot and Ma, 2021). In addition, we previously identified the endoplasmic reticulum protein Reticulon (RTN)-4B/NOGO-B as a new AJC-associated molecule involved in the control of intestinal permeability (Rodriguez-Feo et al., 2015). And besides the cellular elements, the mucus layer constitutes a biologically flexible and efficient film that avoids the direct contact of luminal microorganism and their antigenic components with the IECs (Capaldo et al., 2017). Goblet cells produce and secrete mucins—both free and membrane-anchored—which are the main glycoproteins of the mucus layer. Lack of mucus in mucin-2-deficiency results in increase of both bacterial adhesion to the IECs and intestinal permeability, as well as the enhanced susceptibility to chemical-induced colitis (Grondin et al., 2020). The depletion of mucin is a characteristic feature of ulcerative colitis (UC; McCormick et al., 1990); which has not yet been demonstrated in 12–13-weeks-old IL-10-deficient mice (Makkink et al., 2002).

There are multiple studies that show that the microbiota of patients with CD or UC are different from healthy people or even from their cohabiting relatives (Joossens et al., 2011). However, the study of the microbiota as a cause or consequence in inflammatory disease is complex because there are microorganisms that may have a protective role against inflammation, such as *Faecalibacterium praustnizii*, which is decreased in patients with CD (Rakoff-Nahoum et al., 2004; Sokol et al., 2008) and other known pathobionts that can induce it like certain strains of adherent-invasive *Escherichia coli* (AIEC; Rolhion and Darfeuille-Michaud, 2007). Furthermore, the changes in these microorganisms and the metabolic pathways they are involved in and how this relates to mucosal barrier function and inflammation is still poorly understood.

Early therapeutic interventions could prevent severe intestinal damage also modifying the clinical course of IBD (Berg et al., 2019), however there is scarce knowledge on the pre-clinical events occurring before the clinical symptomatology of intestinal inflammation. On the other hand, it is known that the dysfunction of the intestinal barrier is an early event in the natural history of colitis in IL-10 deficient mice (Arrieta et al., 2009; Keubler et al., 2015), although the mechanisms involved in such disturbance and the relation with dysbiosis or inflammation are still poorly understood. Therefore, the aim of the present work was to systematically characterize the natural history of the colitis in IL-10 deficient mice from 3 to 70 weeks old, focusing on events preceding overt inflammation including microbiota disturbances and intestinal barrier dysfunctions.

## 2. Materials and methods

### 2.1. Mice

C57Bl/6 wild type (WT) and IL-10 deficient (B6.129P2-Il10tm1Cgn/J; IL-10^−/−^) mice were purchased from Jackson (Bar Harbor, MA, United States). All the mice were male and were housed with six mice per cage with 12 h light–dark/day–night cycle, a range of temperature between 23 and 25°C, and humidity of 50% with *ad libitum* access to water and pellet diet (LabDiet, PicoLab, Spain. Reference number 5001) according to the Guide for the care and use of Laboratory Animals (NIH Publication no. 85–23, 1985). The ethical committee on animal experiments of our institution called Comité de Ética de Experimentación Animal (CEEA) approved all experiments under the ethical code PROEX 085-2018. The exclusion criteria for this study were: rectal prolapse or weight loss of 20%. No animal were excluded from the study due to these criteria. The sample size for each strain (WT or IL-10^−/−^) and for each age group was: for 3 weeks of age were used 9 WT and 11 IL-10^−/−^; for 5 weeks, 12 WT and 11 IL-10^−/−^ for 10 weeks, 11 WT and 13 IL-10^−/−^; for 20 weeks, 20 animals in both strains; for 57 weeks, 10 animals in both strains and finally, for 70 weeks, 9 animals in both strains. Mice were euthanized by an overdose of CO_2_ at the age of 3, 5, 10, 20, 57, and 70 weeks, and the entire colon was removed.

### 2.2. Histological scoring

Samples from mice colon were selected for histopathological analysis after methacarm fixation. They were paraffin-embedded, cut in 5 μ sections, deparaffinized, rehydrated, and stained with hematoxylin and eosin (H&E) and scored in a blinded manner. The sample size for this study was five animals for each strain and age group. Histological scoring (HS) was based on a semiquantitative scoring system that graded the following features: extent of destruction of mucosal architecture (0, normal; 1, 2, and 3, mild, moderate, and extensive damage, respectively); presence and degree of cellular infiltration (0, normal; 1, 2, and 3, mild, moderate, and transmural infiltration, respectively); extent of muscle thickening (0, normal; 1, 2, and 3, mild, moderate, and extensive thickening, respectively); presence of crypt abscesses (0, absent or 1, present); and presence of goblet cell depletion (0, absent or 1, present). The scores for each feature were summed with a maximum possible score of 11. An HS < of 3 was considered as normal, whereas between 3 and 7 was considered to correspond to moderate disease, and > of 7 corresponded to severe IBD phenotype.

### 2.3. Myeloperoxidase (MPO) activity

A kit based on a colorimetric reaction was used following manufacturer’s instructions (Sigma, Darmstadt, Germany): MPO catalyzes the formation of hypochlorous acid, reacting it with taurine to form taurine chloroamine. This reacts with the TNB chromophore, converting it to the colorless DTNB product. Finally, a unit of MPO activity is defined as the amount of enzyme that hydrolyzes the substrate and generates taurine chloroamine to consume 1.0 μM of TNB per minute at room temperature (RT).

### 2.4. Preparation of total RNA and real-time PCR

Total RNA from frozen tissue samples was extracted with TissueLyser homogenizer by using 1 ml Tri-pure isolation reagent (Invitrogen, Darmstadt, Germany) according to the manufacturer’s protocol. Contamination with genomic DNA samples was avoided by treatment with DNase (Ambion, Thermo Fisher, Whaltam, MA United States). Reverse transcription was carried out with 500 ng of total RNA by using a Superscript II kit (Bio-Rad Laboratories, Hercules, CA, United States). Specific sets of primers were obtained by using the NCBI/Primer-BLAST designing tool. Animal and cell culture data were corrected respect to their *β-actin* mRNA. List of primers is shown in Supplementary material.

### 2.5. Immunohistochemistry

Tissue 5 μ sections were deparaffinized and rehydrated, boiled in sodium citrate, and blocked in 5% goat serum. The sections were incubated overnight at 4°C with 1:100 polyclonal rabbit anti-RTN-4B/NOGO-B antibody, followed by incubation with reagents from the Vectastain ABC kit (Vector Laboratories, Burlingame, CA, United States) according to manufacturer’s instructions. Staining was developed with DAB (3,3-diaminobenzidine) with Karachi’s hematoxylin as counterstaining. Negative controls were obtained by avoiding the primary antibody as well as by incubation with inactivated anti-RTN-4B/NOGO-B antibody generated as described below.

### 2.6. Intestinal permeability assessment

The amount of dextran labeled with fluorescein isothiocyanate (FITC-dextran) that passes into the blood through the intestinal barrier, after rectal administration, was measured. FITC-dextran was prepared with a concentration of 0.6 mg/g of animal weight, dissolved in 1 × Phosphate buffered saline (PBS). After anesthetizing the mice with sevoflurane, 100 μl of the labeled dextran was administered rectally. After 4 h, under sedation again, 300–500 μl of retrorbital blood were extracted from mice, with the exception of 3 week animals. In this group, it was not possible to assess intestinal permeability due to the insufficient volume of blood obtained in the rest of the groups, blood was collected in microtainer gel serum separation tubes that were centrifuged at 7500 rpm for 15 min. With the serum obtained, the recovery percentage of the FITC-dextran was determined by means of a standard curve with 1/2 dilutions from the reading obtained in the fluorimeter. The excitation/emission spectrum of the probe studied is 485/535 nm.

### 2.7. Microbiota analysis

Feces and colon fragments were immediately frozen at –80°C after collection and processed at the end of the sampling with slowly defrosting at –20°C for 24 h and 4°C for another 24 h, to prevent DNA fragmentation. Both samples (0.2 g) were completely solubilized in 2 ml of water and DNA was obtained from 200 μl aliquots by the QiaAmp kit (Qiagen). Bacterial composition and distribution were determined by PCR amplification of the *16S* rDNA V3-V4 region after massive sequencing (2 × 300 bp) on a MiSeq (Illumina, San Diego, CA, United States) platform. Raw sequence data were deposited in Genbank (BioProject ID PRJNA714289).^1^

Microbial diversity analysis was done with the Quantitative Insights Into Microbial Ecology version 2 (QIIME2) software suite (2019.1 distribution) (Bolyen et al., 2019). Diversity analysis was made using q2-diversity plugin, after samples were rarefied (subsampled without replacement) to 61,458 sequences per sample. We chose this rarefaction depth since it guaranteed robust diversity measures and retained all samples according to rarefaction plot. Diversity analysis comprised an alpha diversity metric (Shannon index, which measure microbiome richness) and a beta diversity metric (Bray–Curtis dissimilarity index, which measure microbiome composition differences). Statistical significance of the differences in mean Shannon index between groups of samples were calculated by Kruskal–Wallis test. To test for differences in microbiome composition between groups of samples, we performed Principal Coordinate Analysis (PCoA) based on the beta diversity Bray-Curtis distance matrix. Permutational multivariate analysis of variance (PERMANOVA) was employed to determine which categorical variables factors explained statistically significant variance in microbiota composition. All statistical tests were conducted *via* q2-diversity plugin from QIIME2. To determine which specific taxa explained beta diversity differences, differential abundance analysis was performed only in variables that yielded statistically significant differences in beta diversity analysis. To do that, linear discriminate analysis effect size (LEfSe) was used for testing taxonomic comparisons (Segata et al., 2011). LEfSe combines the standard tests for statistical significance (Kruskal–Wallis test and pairwise Wilcoxon test) with linear discriminate analysis for taxa selection. In addition to detecting significant features, it also ranks features by effect size, which put features that explain most of the biological difference at top. Alpha value for the factorial Kruskal-Wallis test was 0.05 and the threshold on the logarithmic Linear Discriminant Analysis (LDA) score for discriminative taxa was 2.0.

### 2.8. Colon-derived organoid culture

Organoids were generated with a modificated protocol (Goldspink et al., 2020). Briefly, after sacrifice, colon was extracted and a fragment of 6 cm from cecum was longitudinally opened, washed with cold PBS (without Ca^+2^ and Mg^+2^) and minced in smaller pieces. Tissue fragments were incubated with 30 mM EDTA chelation buffer for 10 min on ice. Next, the EDTA-fragments solution was turned over a Fetal bovine serum (FBS)-precoated Petri dish. Small pieces of colon were taken into a Falcon tube with 10 ml of cold PBS and vigorously shaken to obtain a crypt-enriched fraction. This procedure was repeated until an adequate number of crypts were obtained. The crypt suspension was then filtered through a 70 μm cell strainer and centrifuge al 300 g for 5 min. Pellet was resuspended in culture medium (IntestiCult^™^ medium, StemCell Technologies^©^) and mixed with equal volume of Matrigel (Corning^©^; ratio suspension crypts/Matrigel 1:1). Crypts were then seeded in a pre-warmed 24-well plate in 50 μl droplets per well and allowed to polymerize in an incubator at 5% CO2, 37°C for 30 min. After that, 750 ml of IntestiCult^™^ medium were carefully added in each well and plate was incubated at 37°C, 5% CO2. Culture media were changed every 2–3 days to maintain optimal growth conditions.

### 2.9. Statistical analysis

Data are expressed as means ± SEM. The Mann–Whitney *U*-test was used to compare the nonparametric data and two-way ANOVA followed by Bonferroni’s multiple-comparison test. Only *p* values < 0.05 were considered as statistically significant.

## 3. Results

### 3.1. Animal weight, colonic weight-to-length ratio and histological scoring

**Figure 1:**
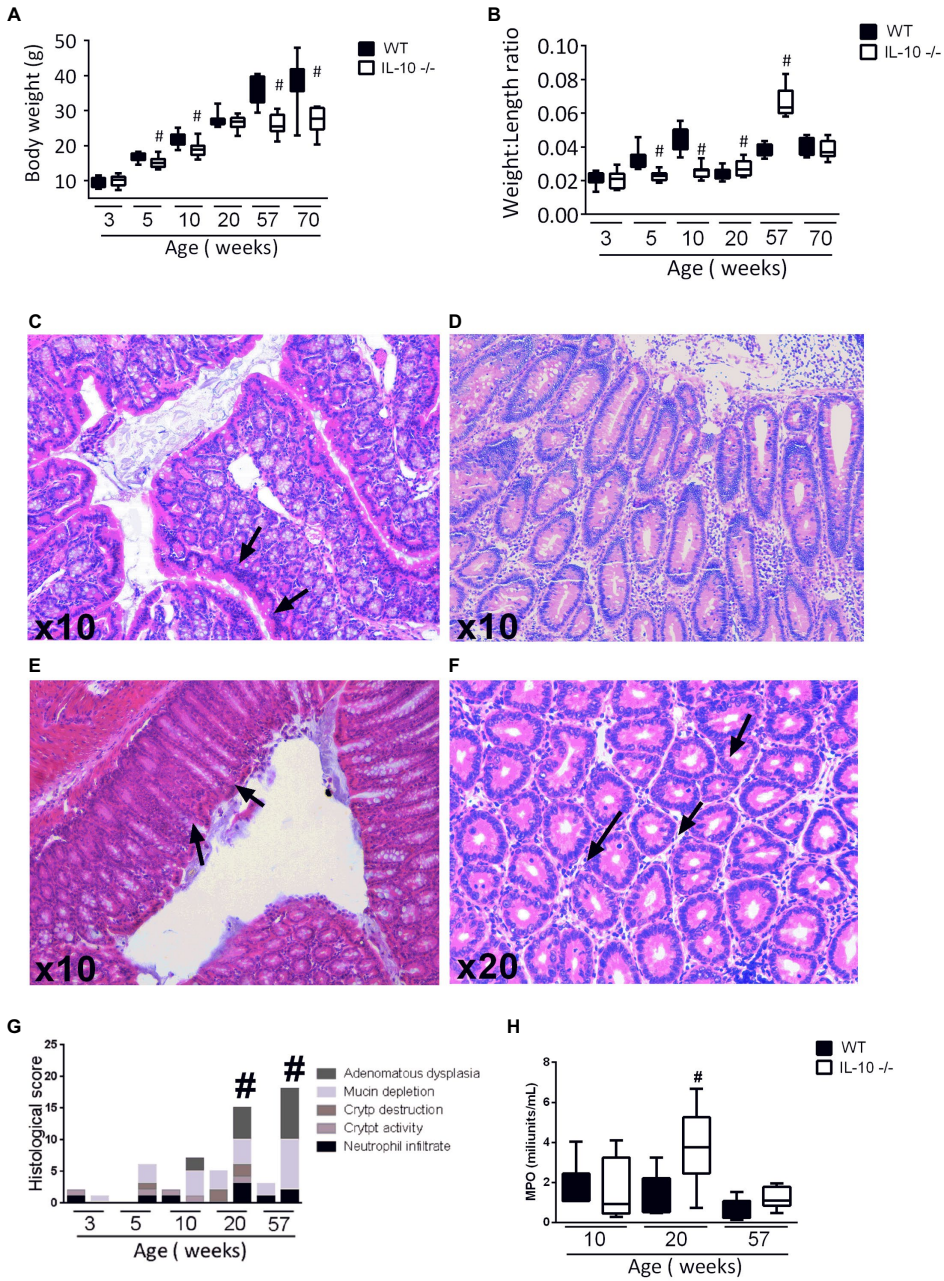
**(A)** Body weight after sacrifice **(B)** Weight to length ratio of the colon of both strains. **(C–F)** Hematoxylin eosin staining in 20-week-old IL-10-deficient mice. Representative images of: inflammatory infiltrate, epithelial stratification, focal erosion, and mucin depletion, respectively. **(G)** Comparison of histological scoring of both strains of all ages (5 animals for each strain and age group were used in this analysis). **(H)** Measurement of the Mieloperoxidase (MPO) enzyme activity by colorimetric reaction at 10, 20, and 57 weeks of age in both strains made in three independent replicates. In all graphs, C57Bl/6 wild type (WT) group was represented in black and IL-10 deficient mice (IL-10^−/−^) group in white. ^#^*p* < 0.05 with respect to wild type (WT).

Compared to WT animals, KO mice showed significant less body weight at 5, 10, 57, and 70 weeks old (Figure 1A). Colonic weight-to-length ratio was significantly higher in KO at 20 and 57 weeks old (Figure 1B). Histological evidence of inflammation—neutrophil infiltration, crypt distortion, goblet cells depletion—reached its maximum degree at 20 weeks-old mice (Figures 1C–1F). Histological scoring was significantly higher in KO animals at 20- and 57-weeks (Figure 1G). Myeloperoxidase activity was evaluated only at the ages showing histological differences in inflammatory infiltrate. Observed values were significantly higher in KO at 20 weeks, with no significant differences at 10- and 57-weeks old mice (Figure 1H).

### 3.2. Colonic expression of pro-inflammatory cytokines and enzymes

**Figure 2:**
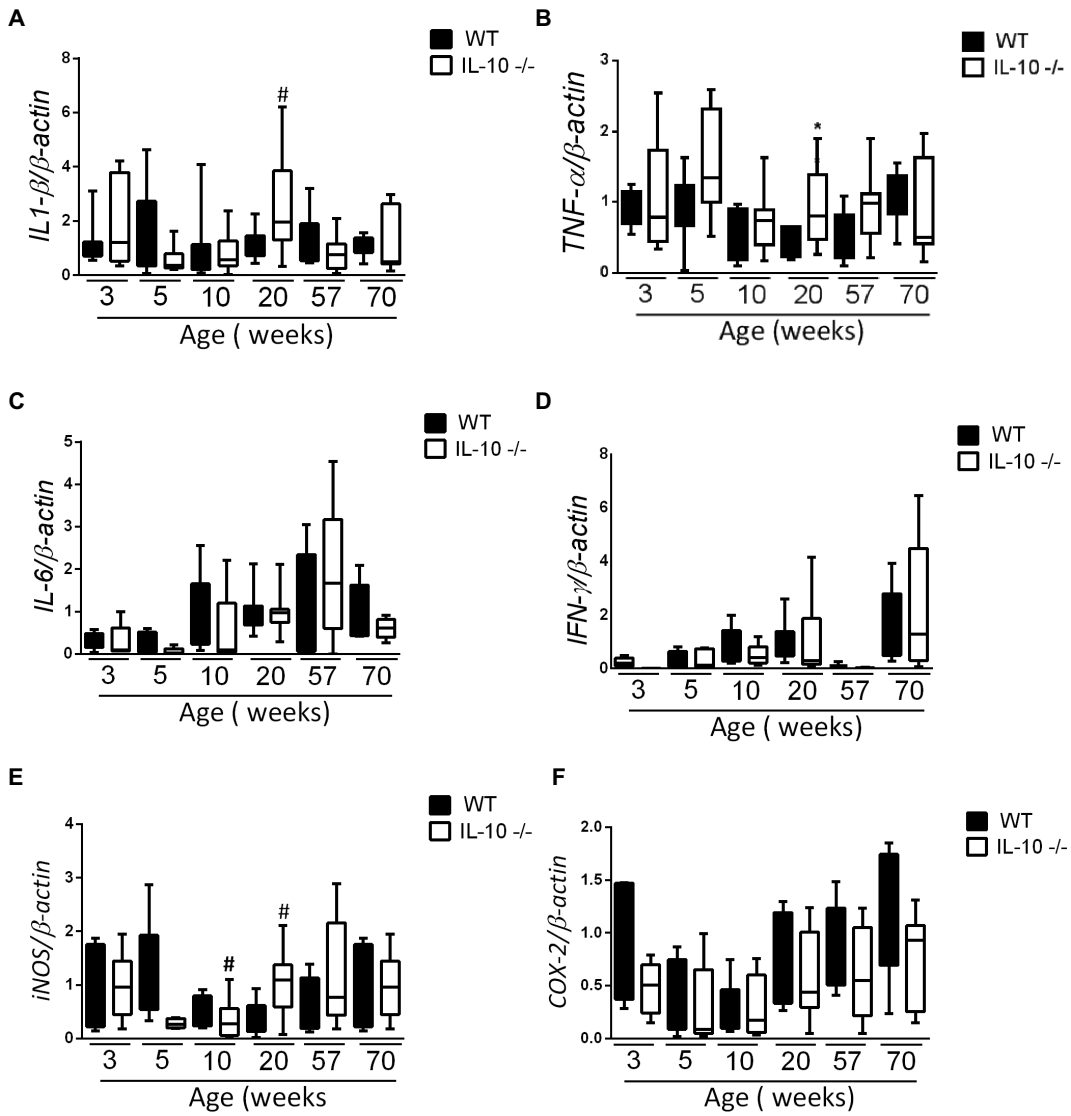
Comparison of *IL-1β* **(A)**, *TNF-α* **(B)**, *IL-6* **(C)**, *IFN-γ* **(D)**, *iNOS* **(E)** and *COX-2* **(F)** gene expression WT and IL-10^−/−^ strains. The normalization was carried out based on the housekeeping gene *β-actin* and three independent replications were made for each marker studied. C57Bl/6 wild type (WT) group was represented in black and IL-10 deficient mice (IL-10^−/−^) group in white. ^#^*p* < 0.05 with respect to WT of the same age.

According with histological findings, *interleukin-1β (IL-1β)* and *Tumor necrosis factor α (TNFα)* mRNA expression in the KO colon at 20 weeks was significantly higher than in their WT counterparts; without significant differences in the remaining samples (Figures 2A,B). We did not find statistically significant differences in the expression of *interleukin-6 (IL-6)* and *interferon γ (IFNγ)* mRNA at any age (Figures 2C,D). Colonic expression of *inducible nitric oxide synthase (iNOS)* mRNA was significantly higher only in KO at 20 weeks (Figure 2E), whereas *ciclooxigenase-2 (COX-2)* mRNA expression was homogenous in both groups and sampling times (Figure 2F).

### 3.3. Intestinal barrier function and adhesion molecules mRNA expression

**Figure 3:**
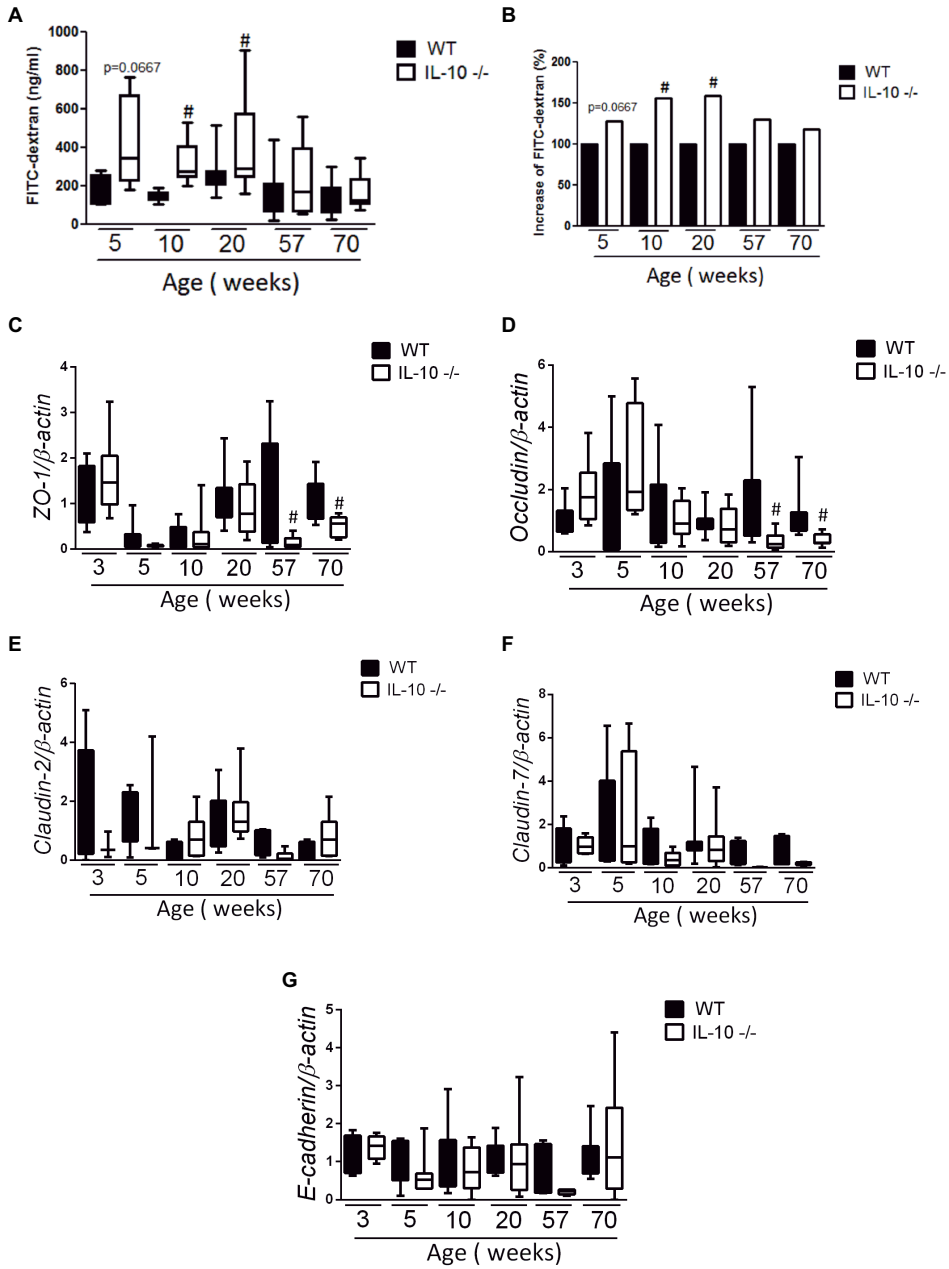
*In vivo* permeability and gene expression of adhesion molecules. Measurement of fluorescein isothiocyanate (FITC-dextran) fluorescence in serum after passive diffusion through the intestinal epithelium and after rectal administration (*n* = 6 for each strain and age); absolute values **(A)** and percentage increase **(B)**. Comparison of gene expression of adhesion molecules *ZO-1* **(C)**, *Occludin* **(D)**, *Claudin-2* **(E)**, *Claudin-7* **(F)** and *E-cadherin* **(G)**. The normalization was carried out based on the housekeeping gene *β-actin* and three independent replications were made for each marker studied. C57Bl/6 wild type (WT) group was represented in black and IL-10 deficient mice (IL-10^−/−^) group in white. ^#^*p* < 0.05 to respect to WT of the same age.

Compared to WT, the colonic permeability to FITC-dextran was significantly higher in KO mice from week 10 (Figures 3A,B); but equivalent at 57- and 70-weeks. The expression of *ZO-1* (Figure 3C) and *Occludin* (Figure 3D) mRNA was significantly lower in KO at 57- and 70-weeks, whereas no significant differences were found in the expression of *Claudin-2*, *Claudin-7* or *E-cadherin* mRNA (Figures 3E–G).

### 3.4. Mucin content and endoplasmic reticulum stress (ERS) markers

**Figure 4:**
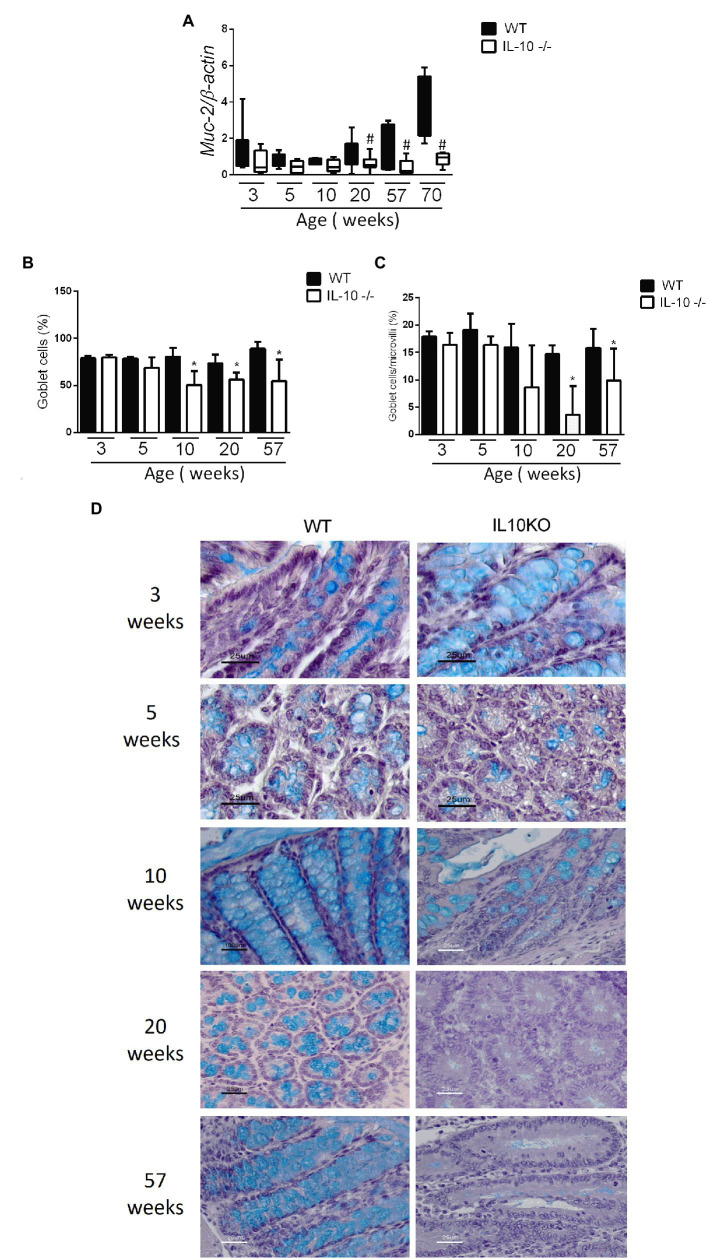
**(A)** Comparison of gene expression of *MUC-2* gene in colon of WT and IL-10^−/−^ mice in all evaluated ages. ^#^*p* < 0.05 to respect to WT of the same age. The count of total goblet cells **(B)** and goblet cells per microvillus **(C)** was performed blindly by two independent researchers from images obtained with ACT-1 software. C57Bl/6 wild type (WT) group was represented in black and IL-10 deficient mice (IL-10^−/−^) group in white. ^#^*p* < 0.05 to respect to WT of the same age. **(D)** Histological sections of the colon stained with alcian blue and hematoxylin in WT and IL-10^−/−^ at 3, 5, 10, 20, and 57 weeks of age.

**Figure 5:**
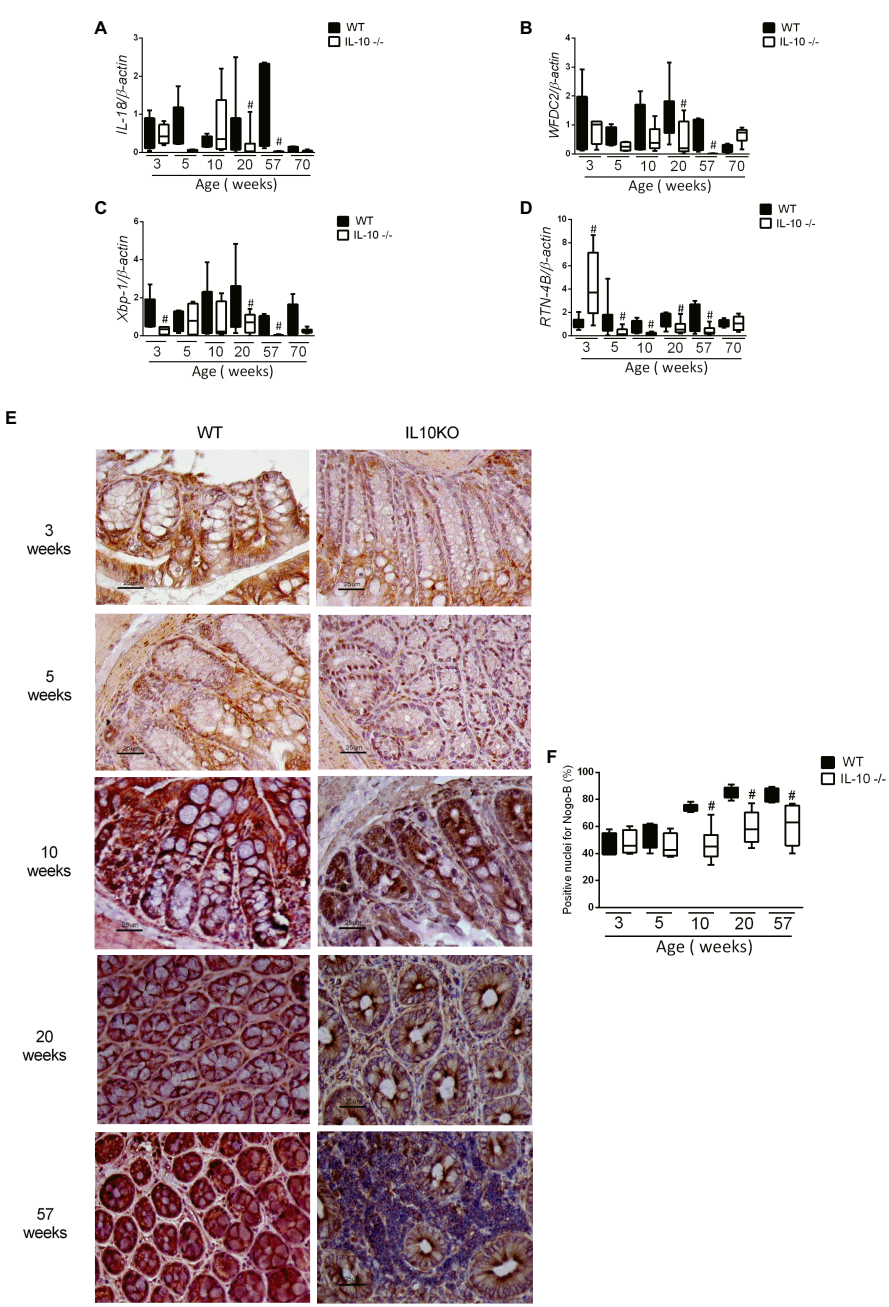
Analysis of gene expression by qPCR of *IL-18* **(A)**, *WFDC2* **(B)**, *Xbp1* **(C)**, and *RTN-4B/NogoB* **(D)** in the colon of WT and KO mice in all evaluated ages. C57Bl/6 wild type (WT) group was represented in black and IL-10 deficient mice (IL-10^−/−^) group in white. #p < 0.05 with respect to WT of the same age. **(E)** Histological sections of the colon of WT and IL-10^−/−^ mice at 3, 5, 10, 20, and 57 weeks of age labeled with RTN-4B antibody and revealed with DAB (*n* = 6 for each strain and age). **(F)** RTN-4B positive nuclei count from images acquired in ACT-1 software in blindly and independent manner.

The expression of the *MUC2* gene in colon was significantly lower in KO at 20, 57 and 70 weeks-old (Figure 4A). In the same way, the content of mucin in the epithelium, estimated by means of positive goblet cells in the alcian blue staining, were significantly lower for IL-10 deficient mice starting at week 10, as well in 20-and 57-weeks old mice (Figures 4B–D). In order to in-depth explore the mechanisms associated with mucin depletion in the colon of this murine model of colitis, we next analyzed expression of *interleukin-18 (IL-18)* and *WAP Four-Disulfide Core Domain 2 (WFCD-2)* genes which was significantly lower for KO at 20 and 57 weeks old, without statistical differences at early ages (Figures 5A,B).

In addition, early (3 weeks-old) decrease of colonic *X-box-binding protein (Xbp-1)* mRNA expression was observed in KO (Figure 5C); as well as the *RTN-4B/NogoB* expression was significantly lower in KO from 5 to 57 weeks (Figure 5D). Immunohistochemistry of RTN-4B/NogoB confirmed those findings (Figures 5E,F).

In order to explore possible intrinsic defect on mucin synthesis in the colonic epithelium, we took advantage of the use of colonic organoids from 20-weeks old mice: no significant differences were found in the mRNA expression of *MUC-2* or *Xbp-1* between both groups of mice—derived organoids (data not shown).

### 3.5. Gut microbiota composition in feces and colonic mucosa

*16S* rDNA determinations were performed in at least six mice per group and age, except at 3 weeks where sampling was three animals per group. After the initial quality analysis, 30 millions of readings were adequately identified up to the taxonomic category of genus. Alpha-diversity, as measured by the Shannon index, only detected significant differences at 20 weeks of age in colonic mucosal samples, with higher diversity values for KO mice (Supplementary material).

**Figure 6:**
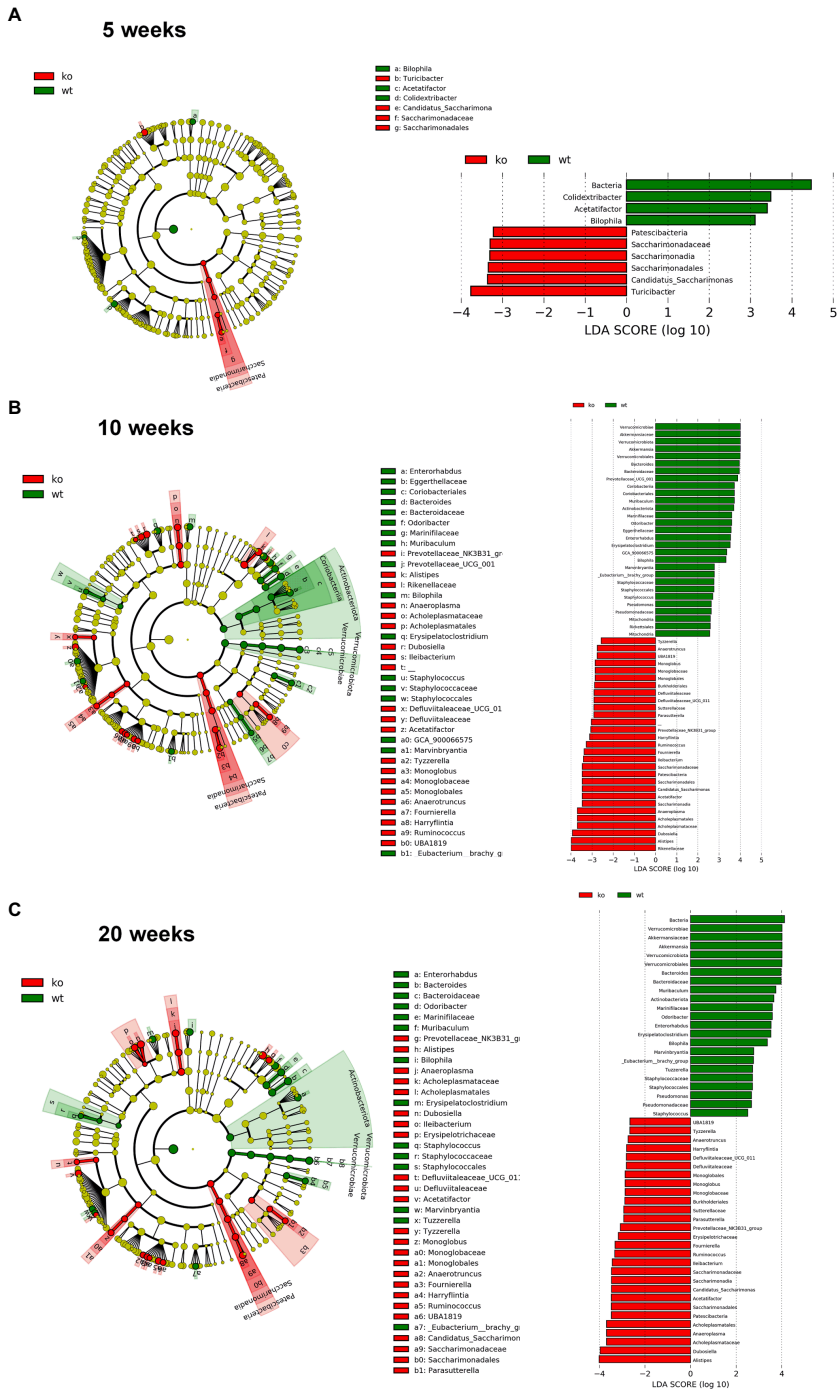
Comparison of the differences in the composition of the microbiota of C57Bl/6 wild type (WT) and IL-10 deficient mice (IL-10^−/−^) IL-10^−/−^ from colonic mucosa samples with linear discriminant analysis effect size (LEfSe) at ages 5 **(A)**, 10 **(B)** and 20 weeks **(C)**. Significant differences are considered from 2.0 on the logarithmic scale, both positive and negative. The cladogram indicates the importance of the changes in the bacterial composition for the rest of the populations of the sample.

**Figure 7:**
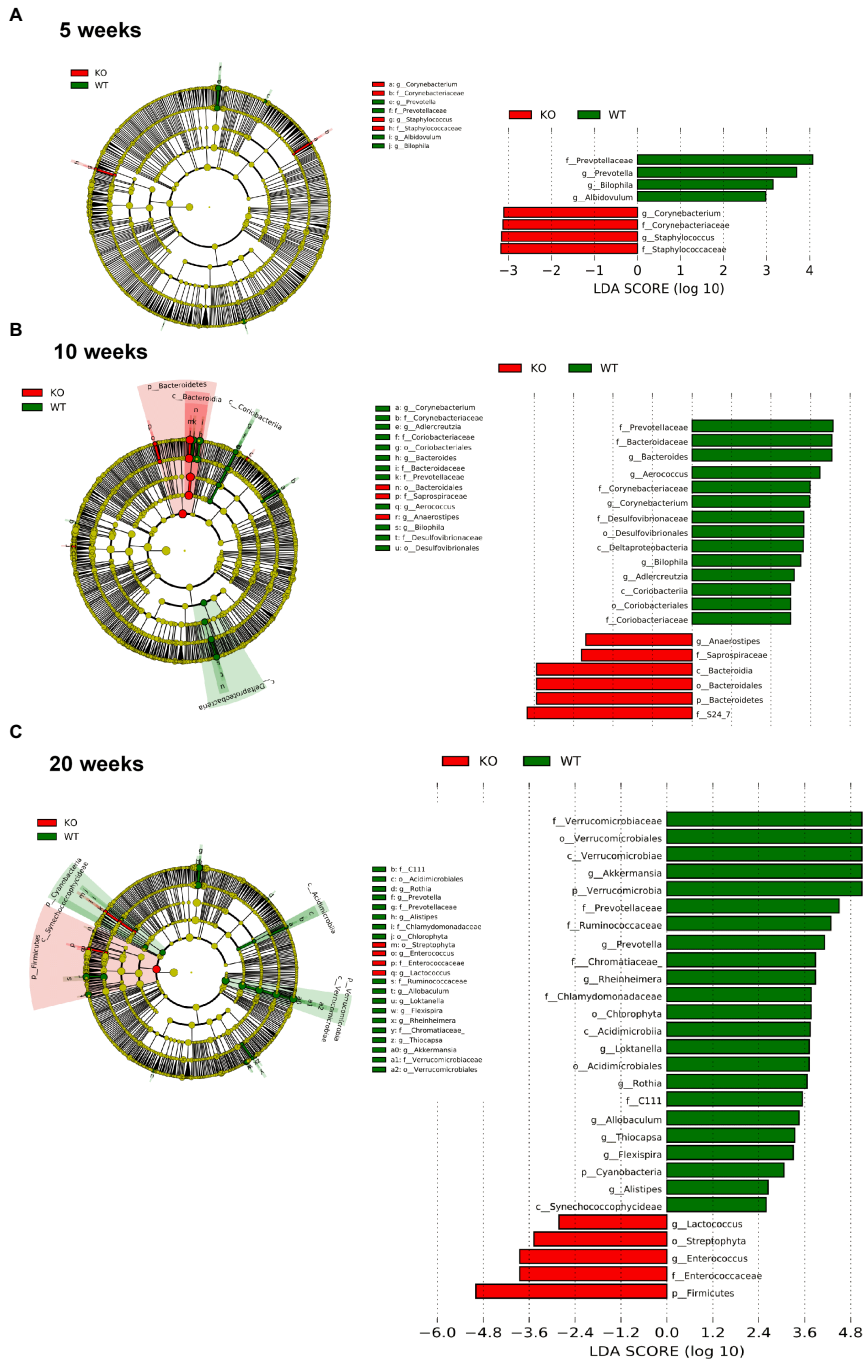
Comparison of the differences in the composition of the microbiota of C57Bl/6 wild type (WT) and IL-10 deficient mice (IL-10^−/−^) IL-10^−/−^ from fecal samples with linear discriminant analysis effect size (LEfSe) analysis at ages 5 **(A)**, 10 **(B)** and 20 weeks **(C)**. Significant differences are considered from 2.0 on the logarithmic scale, both positive and negative. The cladogram indicates the importance of the changes in the bacterial composition for the rest of the populations of the sample.

However, significant differences on beta-diversity measured by Bray–Curtis and LeFSe algorithms started at 5 weeks in feces and at 3 weeks in colonic mucosa (Supplementary material), being deeper at 10 weeks and even deeper at 20 weeks. At 20 weeks in both feces and colonic mucosa, the most notorious feature was a considerable decrease on *Akkermansia* (phylum *Verrucomicrobia*; Figures 6, 7). As feces contains DNA from the whole intestinal tract, some differences respect to the colonic mucosa was observed, but in both samples the *Akkermansia* depletion was notorious. Changes in gut microbiota composition observed at 20 weeks persisted on 57 weeks-old animals (data not shown). In addition, another common characteristic between the microbiota from the mucosa and feces is the loss of biodiversity that can be seen with age. Specifically, the most notable changes in the feces are the loss of some families and genera in the IL-10^−/−^ strain, such as *Albidovulum* and *Prevotella* (5 weeks old) or *Adlercruztia equolifaciens* (10 weeks old). However, at 20 weeks of age, families such as *Enterococcaceae* appear in IL-10^−/−^ mice and do not seem to be significantly present in WT. Regarding the changes observed with the LeFSe analysis in the mucosal microbiota, there is loss of families such as *Prevotellaceae* (10 weeks) or *Bacteroideaceae* (20 weeks).

## 4. Discussion

Enhanced intestinal permeability seems to be a key pathophysiological phenomenon of human IBD (Kevans et al., 2015) and several animal models of IBD such as IL-10 deficient mice (Arrieta et al., 2009). Intestinal barrier dysfunction results in bacterial translocation and enhanced uptake of luminal antigens which, consequently, are associated with maintained stimulation of *lamina propria* and submucosa immune cells, and finally, with chronic inflammation. The results reported herein demonstrates that intestinal barrier dysfunction is an early event in the natural history of colitis in IL-10 deficient mice, preceding the development of overt inflammation, as it seems to occur in human CD (Turpin et al., 2020).

It is well known that proinflammatory cytokines such as IFNγ and TNFα, and soluble mediators such as nitric oxide (NO) and reactive oxygen species (ROS) cause alterations and/or disassembly of intercellular junctions, leading to intestinal barrier dysfunction (Buckley and Turner, 2018; Mu et al., 2019). Nevertheless, our results strongly suggest that the increase of the intestinal permeability occurs in the absence of overt inflammation or even enhanced expression of the aforementioned pro-inflammatory markers; therefore, it is tempting to speculate that the intestinal barrier dysfunction is not an epiphenomenon related with subepithelial inflammation but a primary pathophysiological event. Supporting this hypothesis, genetic studies have demonstrated a relationship between 3020insC mutation of *CARD15*/*NOD2* gene and higher mucosal permeability among first-degree relatives of CD patients (Buhner et al., 2006); moreover, an allelic variant of *DLG5* (*Drosophila discs large homolog* 5) gene, that codifies a intracellular scaffold protein involved in the maintenance of epithelial barrier integrity, is associated with a higher risk of CD and UC development (Stoll et al., 2004). In addition, it has been recently demonstrated that increased intestinal permeability in asymptomatic first-degree relatives of CD patients is associated with later development of the disease (Turpin et al., 2020).

Intestinal barrier comprises cellular and extracellular elements (Turner, 2009). Intestinal epithelial cells constitute the main cellular element of the intestinal barrier, and tight junctions between contiguous intestinal epithelial cells constitute the rate-limiting seal of the paracellular barrier pathway. In the present study, we did not find correlation between an increased permeability and the expression of intercellular junctions’ molecules. Nevertheless, changes in protein expression and distribution have not been explored and deserves further investigation. But *RTN4-B/NOGO-B* mRNA and protein levels are significantly lower in the colon of 5-, 10-, 20-and 57-weeks old KO mice. We previously showed that RTN-4B/NOGO-B is an apical junction complex-associated molecule in the surface intestinal epithelium, which expression is decreased in the inflamed colon of 20-weeks old IL-10 deficient mice and CD patients (Rodriguez-Feo et al., 2015).

Among the extracellular components, Muc2 mucin synthesized and secreted in the gut by goblet cells is a key player of the intestinal barrier function (Kang et al., 2022). Altered Muc2 synthesis and, consequently, aberrant mucin assembly induces endoplasmic reticulum stress and promotes colonic inflammation in mice (Heazlewood et al., 2008). In this sense, IL-10 could enhance intestinal barrier function by inducing the production of mucus *via* suppression protein misfolding and endoplasmic reticulum stress (ERS) in goblet cells (Hasnain et al., 2013). Thus ERS-induced mucin depletion seems to be a relevant pathogenetic mechanism of colitis in IL-10 deficient mice; our results demonstrating a significantly reduced number of goblet cells and expression of *MUC-2, IL-18, WFDC2* and *Xbp-1* in IL-10 deficient mice at young ages support this hypothesis. In addition, ERS also might be involved in the early decrease of the endoplasmic reticulum protein RTN-4B/NOGO-B expression and distribution described in the present work. RTN4-B/NOGO-B is a structural protein of the endoplasmic reticulum that participates in the maintenance of ER tubular shape and functions (Rodriguez-Feo et al., 2015).

Gut microbiota seems to be a main trigger of the aberrant immune response that characterizes human and murine IBD (Putignani et al., 2016), and the presence of antibodies against bacterial antigens in healthy individuals is associated with later development of CD (Torres et al., 2020). In the present work, we have shown that bacterial composition modifications are an early event in IL-10 deficient mice, starting at week 5 of age—before the development of overt or even subtle colonic inflammation. In this context, Leibovitzh et al. (2022), have recently shown gut microbiota alterations in healthy first-degree relatives of CD patients, associated with intestinal permeability dysfunction. The decrease in the genus *Prevotella* was observed in the feces and mucosa of IL-10-deficient mice. This genus has been defined as protective in the development of IBD, due to its role as a commensal microbiota that intervenes in the degradation of fibers and organic compounds (Kovatcheva-Datchary et al., 2015; Azimirad et al., 2022). In addition, the considerable decrease on *Akkermansia* abundance was corroborated in both feces and colonic mucosa and is presumably a direct consequence of the drastic reduction of the mucus layer. Interestingly, it has been recently shown that Muc2 mucin is essential in the maintenance of a “healthy” microbiota in mice, protecting them against chemical-induced colitis (Leon-Coria et al., 2021). However, other authors have pointed to *A. muciniphila* to promote colitis in a genetically susceptible mouse model (Seregin et al., 2017). These results might be due to specific-pathogen free housing conditions. IL-10 deficient mice used in this work were kept in conventional housing conditions since the development of colitis is microbiota-dependent (Sellon et al., 1998).

Future researches should be achieved to better understand the correlation between intestinal permeability and adhesion molecules expression, including some other TJs families like nectins, catenins, desmins, etc. Mucin depletion observed in the colon of IL-10 deficient mice seems to be related to ER-stress. It would be interesting to clarify the relation with ER stress and other mechanisms related to translation and folding of highly glycosylated proteins such as mucins. Regarding this, proteomic assays of ER enriched fraction could be helpful to unravel the underlying mechanism of altered mucin secretion. In this study, relevant changes in microbiome composition have been observed in IL-10 deficient mice. In-depth microbiota analysis including transcriptomic and metabolomic studies would be interesting tools to highlight functional implications of these alterations.

## 5. Conclusion

Mucin depletion and microbiota refitting—probably related with ERS—in the colon of IL-10 deficient mice are associated with early intestinal barrier dysfunction and precede overt gut inflammation in this animal model of chronic intestinal inflammation. Intriguingly, we demonstrate significant weight loss in IL-10 deficient mice at early ages without evidence of histologic inflammation, and this point deserves further investigation. Characterization of the chronological course of murine colitis, and identification of novel mechanisms involved in the disturbed intestinal epithelial barrier function in experimental models might provide a better understanding of IBD pathophysiology and natural history.

## Data availability statement

The datasets presented in this study can be found in online repositories. The names of the repository/repositories and accession number(s) can be found at: https://www.ncbi.nlm.nih.gov/genbank/, PRJNA714289.

## Ethics statement

The animal study was reviewed and approved by Comité de Ética Animal de Experimentación Animal (CEEA) del Instituto de Investigación Sanitaria Gregorio Marañón.

## Author contributions

BL-C, MP, and LM conceived and designed the research, interpreted the results of experiments, and prepared figures. BL-C, MP, FB-A, JG, and JL performed experiments. BL-C, MP, MP-A, IP, MF-A, YG-N, and LM analyzed data. BL-C, MP, MF-A, JM-B, IM-J, RB, and LM edited and revised the manuscript. BL-C and LM drafted the manuscript and approved final version of the manuscript. All authors contributed to the article and approved the submitted version.

## Funding

This work was supported by grants from Instituto de Salud Carlos III (grant numbers PI16/02096 and PI19/01746 to LM).

## Conflict of interest

The authors declare that the research was conducted in the absence of any commercial or financial relationships that could be construed as a potential conflict of interest.

## Publisher’s note

All claims expressed in this article are solely those of the authors and do not necessarily represent those of their affiliated organizations, or those of the publisher, the editors and the reviewers. Any product that may be evaluated in this article, or claim that may be made by its manufacturer, is not guaranteed or endorsed by the publisher.

## Footnotes

^1^ https://dataview.ncbi.nlm.nih.gov/object/PRJNA714289?reviewer=dfivjqc675ru7cjcjv9656uq4m

## Supplementary material

Supplementary Figure 1 Sample size (*n*) used for each mouse strain and age.

Supplementary Figure 2 List of primers used in all the RT-PCRs of the study.

Supplementary Figure 3 α- and β-diversity: microbiota study from fecal samples. The sample size for all the microbiota experiments and for each strain and age is 6, except in 3 weeks of age, where it is 3. Shannon index representing alpha diversity for both strains and ages (**A**). Bray-Curtis that collects the beta diversity for both mouse strains and all ages (**B**) and representation of the samples whose differences are significant (**C**).
